# Centenarians, Supercentenarians: We Must Develop New Measurements Suitable for Our Oldest Old

**DOI:** 10.3389/fpsyg.2021.655497

**Published:** 2021-04-07

**Authors:** Joyce Shaffer

**Affiliations:** Department of Psychiatry and Behavioral Sciences, University of Washington, Seattle, WA, United States

**Keywords:** centenarians, supercentenarians, brain plasticity, neuroplasticity, positive psychology, neurogenesis, healthy aging, cognitive

## Introduction

Researchers and clinicians need to develop measurements that capture the nuances of the many senior successes found in centenarians and supercentenarians in order to empower people to improve healthspans for the duration of extreme lifespans. Only after these measurement tools are created can we accurately assist our oldest old in fostering further development. Marian Diamond, the mother of neuroplasticity, opined that her evidence was applicable in humans; with the solitary change of adding “TLC” to the research protocol, her rats continued “Enriching Heredity” throughout the equivalent of 90 human years; this could be the first proof of the power of positive psychology (Diamond, 1988, 2001). The Flynn effect (Flynn, 2018) found the 20th century was “dominated by massive IQ gains from one generation to another” as documented in “at least 34 nations.” Neuroscience provides evidence-based interventions to “prevent, delay onset, and/or reverse” cognitive decline and dementia (Ball et al., 2002, 2013; Mahncke et al., 2006) and to enhance brain architecture and function (Diamond, 1988, 2001; Pereira et al., 2007; Angevaren et al., 2008; Larson, 2008; Baker et al., 2010; Lojovich, 2010; Erickson et al., 2011, 2014; Jessberger and Gage, 2014; Nagamatsu et al., 2014; Niemann et al., 2016; Ryan and Nolan, 2016; Shaffer, 2016; Burzynska et al., 2017; Edwards et al., 2018). This article reviews research showing that healthy aging without dementia is achievable (Andersen-Ranberg et al., 2001; Perls, 2004; Jopp et al., 2016; Qiu and Fratiglioni, 2018) even with lesions postmortem (Mizutani and Shimada, 1992) and urges development and application of measurements suitable for centenarians and supercentenarians which could inform development of evidence-based interventions across the extreme lifespan.

Search efforts were primarily within PubMed using combinations of keywords listed above and names of prominent researchers in the field. Reference lists in articles and related reviews were searched. Studies reviewed met rigorous scientific standards, were review articles or random control trials. Periodic searches of this nature were conducted between 2016 and 2021.

## Senior Successes

Centenarians and supercentenarians can maintain extraordinary cognitive and physical performance. One example is the 118-year-old woman whose neuropsychological tests found slight improvement in her cognitive functioning over 6 months (Ritchie, 1995). Another example is the 112-year-old woman whose score of 27 on the MMSE was the maximum possible without vision. At 113 years her scores were “still within the normal range for healthy older adults” of 60 to 75 years and, after death at 115, there was “no significant atherosclerosis, and her brain no vascular pathology, only a slight amount of tau pathology.” (den Dunnen et al., 2008). Both women had visual and hearing impairments. Studies suggest that senile dementia can be seen as “age-related” because it occurs within specific ages, “rather than as an ‘age-related’ disorder (that is, caused by the aging process itself)” (Ritchie and Kildea, 1995). Dutch centenarians who scored 26 or above on the MMSE maintained their high level of cognitive performance 2 years later (Beker et al., 2019, 2020). It is significant that measurement tools have had to be modified because of their sensory and physical limitations. It is noteworthy that their test results could only be compared to norms established on younger individuals.

The Nun Study (Snowdon, 2003) combined annual evaluations of cognitive and physical function with postmortem neuropathologic evaluations. The “gold standard” for healthy aging, Sister Matthia, continued to be “happy, productive, and vivacious” to the end of her life just weeks short of 105, having “enjoyed more than 100 years of dementia-free life.” Sister Marcella's “life, cognitive abilities and longevity were extraordinary;” at 100 her Mini-Mental Status Examination score was 28/30. At postmortem “she had a remarkably clean, large brain” when she died at 101.

Of twenty-seven centenarian brains, there were three “supernormal” centenarians who had retained good mental functioning; in their neuropathological findings “no apparent senile changes or ischemic lesions” were found. The post-mortem findings of the 27 brains were “not fundamentally different” than findings of brains sof less elderly (Mizutani and Shimada, 1992).

Although percentages varied, there were centenarians with no dementia in many countries: 37% in Denmark (Andersen-Ranberg et al., 2001); 54% in Italy (Motta et al., 2008); 21% in New England (Silver et al., 2001); the Fordham Centenarian Study found no, or very few, cognitive limitations in 119 centenarians (Jopp et al., 2016); 188 Australian centenarians had good cognition (Richmond et al., 2012); and a review of 11,084 electronic records in the United Kingdom found only 12% of women and 6% of men diagnosed with dementia (Hazra et al., 2015). Almost 57% of 228 Chinese individuals aged 100-112 were independent (Huang et al., 2020). The Framingham Heart Study (Satizabal et al., 2016) found a decline in incidence of dementiass across three decades.

In 57 dementia-free centenarians, the finding that “stronger functional connectivity between right frontoparietal control network,” and a stronger functional connectivity compared to subjects aged 76 to 79, demonstrated a more intact bilateral neuronal efficiency. This “may relate to the resistance to cognitive decline in our cohort of dementia-free near-centenarians and centenarians who can be considered as successful agers” (Jiang et al., 2020). One theory about how some remain cognitively efficient proposes that, during their prenatal and neonatal life, neuron selection for organizing specific areas of the cortex was more accurate. Thus, less neurons which were vulnerable to degeneration promoted by amyloid deposits were able to survive. This left a healthier and stronger array of neurons for cortical neural circuits which could resist age-related changes (Bugiani, 2020).

A compression of morbidity has been documented (Andersen et al., 2012; Richmond et al., 2012; Sebastiani et al., 2012, 2019; Hashimoto et al., 2019). Thriving more than a century in good health with compressed morbidity is “a good model of successful aging” (Hashimoto et al., 2019). Among the reasons exercise remains important is that it increases brain factors which might play a role in “reversing brain aging” and “improve cognitive functioning” (Baker et al., 2012; Baker, 2015; Horowitz et al., 2020). Chinese elders with MCI appreciated cognitive gains, less depression and better balance after 12 weeks of square dancing (Wang et al., 2019). After 18 months of dancing, participants had improved balance as well as increased volume of the hippocampus (Rehfeld et al., 2017) as measured by MRI as compared to individuals whose physical fitness routine was conventional. Increased plasma BDNF was found in dancers whose attention and spatial memory were improved (Rehfeld et al., 2018).

Robert Marchand was the first centenarian to document improved performance and health (Billat et al., 2017). After setting a world record for cycling at age 101, with 2 years of training he beat by 10% his previous record for speed and improved his VO2max to the same range that would classify men aged 42 to 61 as physically fit. Along with other “Olympic” centenarians the message is clear that it is never too late for beginning and benefitting from exercise (Sanchis-Gomar et al., 2014; Lepers et al., 2016).

Healthcare success in supercentenarians includes the 111-year-old woman who walked out of the hospital after hip arthroplasty (Wu et al., 2020). The use of convalescent plasma to successfully treat a centenarian who had COVID-19 (Kong et al., 2020) is another centenarian healthcare success.

The Okinawans might be “the world's healthiest and longest lived people” (Willcox et al., 2017); “over 1,000 centenarians” were examined between 1975 and 2015; their healthy lifestyle included a nutritionally dense, low calorie diet that was high in vegetables including sweet potatoes and soy; they remained physically active. In Washington State USA, factors associated with living to 100 included living in a neighborhood that was walkable (Bhardwaj et al., 2020). Exercise increases brain factors which might play a role in “reversing brain aging” and “improve cognitive functioning” (Horowitz et al., 2020).

Taiwanese being independent was associated with cognitive preservation whereas Koreans had better cognitive preservation living alone (Hsieh et al., 2020). Frequency of children visiting helped maintain cognitive efficiency in Chinese elders (Yin et al., 2020). Decreased risk for dementia has generally been associated with increasing years of education (Jopp et al., 2016; Satizabal et al., 2016; Langa et al., 2017; Holstege et al., 2018).

## Measurement Issues

The Mini Mental Status Evaluation (MMSE) can assess language, attention, concentration, and orientation (Folstein et al., 1975). It is among the most frequently used measurement tools. The Montreal Cognitive Assessment (MOCA) might be more sensitive to mild changes in cognitive functioning and a telephone adaptation of the MOCA seeks to accommodate the oldest old too distant from testing facilities as well as those affected by a pandemic (Katz et al., 2021).

Whether using these measurement tools or using tests that take about 5 min Mini-cog, AD8, FAQ), tests taking up to 10 min including the SLUMS and TICS, or self-administered tests, all would be subject to the same limiting factors which would likely influence the accuracy of test results. As the case studies emphasized above, these factors include but may not be limited to sensory, physical, and educational differences in this age cohort. These elders as well as their proxies can have limited awareness of, as well as reservations about expressing, estimates of level of functioning. Although cognitive status of centenarians and supercentenarians is a major factor in their well-being and functioning, it is difficult to evaluate with current measures (Arosio et al., 2017). Norms for these oldest old do not exist. That makes it equally difficult to determine the value of evidence-based interventions studied in younger populations (Patnode et al., 2020).

There are simultaneously gifts and challenges within the prediction that the world “soon will have more older people than children and more people at extreme old age than ever before.” The gifts include the growing possibilities for longitudinal studies in centenarians and supercentenarians such that we can develop measurement tools specific to their sensory, physical, social, educational, and cultural situations. Challenges will include determining which of the evidence-based interventions currently available could be beneficial to our centenarians and supercentenarians. For example, much useful field of view (UFOV) research (Edwards et al., 2018) showed that “UFOV training enhanced neural outcomes, speed of processing, and attention” as well as far transfer to such everyday functioning as prolonged and safer driving; it is significant that “improvements on the trained skills endured across 10 years.” Research could evaluate the benefits of UFOV training for centenarians and supercentenarians since it is accepted that genes do not dictate; the research on neuroplasticity and aging shows neuroplastic adaptability endures. The nature-nurture interaction includes change as shown in research finding differences in genetic expression of monozygotic twins at age 50 (Fraga et al., 2005).

Regenerative medicine could gain relevance; researchers reported “spontaneous reversal of cellular aging in supercentenarian donor cells” (Lee et al., 2020) obtained from the NIA longevity collection of a female who “had an exceptional health record with no reported history of typical age-related diseases except for cataracts.” The researchers concluded that “neither short telomere length nor extreme natural human aging in the supercentenarian donor are absolute barriers to reprogramming both developmental state and cellular age.”

## We Must Develop Measurements and Interventions for Centenarians and Supercentenarians

Much has been learned about thriving more than a century, yet we have only begun the essential measurement of the nuances of the multifaceted successes of our centenarians and supercentenarians. Development of age-appropriate measurement must precede the quest for evidence-based interventions to promote maximum cognitive, physical, social, cultural, and emotional functioning in health and wellness across more than a century with a healthspan for the duration.These interventions must include “TLC” to capture how positive psychology induced “Enriching Heredity” with a 50% increase in lifespan that included neuroplastic gains in rats to the equivalent of 90 human years (Diamond, 1988, 2001). Interventions also must include speed of processing influences as demonstrated by the UFOV data (Edwards et al., 2018). Interventions such as in the “Finnish Geriatric Intervention Study to Prevent Cognitive Impairment and Disability (FINGER)” which are multidomain in lifestyle choices (Ngandu et al., 2015) must be included and assessed in our oldest old.

“Use them and they will grow” approximates the new mantra to empower people to maximize positive neuroplasticity in ways that simultaneously enhances other components of health with the goal of having a healthspan for the duration of the lifespan. The finding that monozygotic twins have different genetic expressions at age 50 (Fraga et al., 2005) encourages further clinician-researcher collaborations on how to maximize “Enriching Heredity” with influences on inheritance-environment epigenetic spaces where both anabolic and catabolic activities might be influenced in a positive direction.

Prevention is far preferable to intervention and care; it could increase the wisdom of the aged. Lancet's Commission (Livingston et al., 2017) recommending being “ambitious about prevention” with a focus on interventions that “might have the potential to prevent a third of dementia cases” is timely; it is also urgent. Surely the evolving research on the successes of centenarians and supercentenarians expands our database and our models of success in maximizing potential for a healthspan that approximates extreme lifespan. However, it is urgent that we expedite development of measurements for several aspects of functioning so that we could (1) celebrate more accurately the senior successes; (2) revisit the perspective of Ramscar et al. (2014, 2017) about the “myth of cognitive decline;” (3) appreciate more benefit from the wisdom of the aged; AND 4) assess potential value of evidence-based interventions in people who thrive for more than a century.

## Author Contributions

The author confirms being the sole contributor of this work and has approved it for publication.

## Conflict of Interest

The author declares that the research was conducted in the absence of any commercial or financial relationships that could be construed as a potential conflict of interest.
